# Tradeoffs for bluegill sunfish turning at different rates

**DOI:** 10.7717/peerj.21161

**Published:** 2026-04-28

**Authors:** Andrew David Clark, Eric D. Tytell

**Affiliations:** Department of Biology, Tufts University, Medford, MA, United States of America

**Keywords:** Swimming, Kinematics, Moment of inertia, Torque, Momentum, Fish

## Abstract

When fish turn using their caudal fins, they face a tradeoff. To turn rapidly, they must produce high torque or reduce their rotational moment of inertia or both, but these two things may pose opposite constraints. The moment arm for torque from forces from the caudal fin is highest when the body is straight, but the moment of inertia is lowest when the body is bent. Torque depends on the distance of body segments from the center of mass, but moment of inertia depends on this distance squared. Therefore, we hypothesized that fish would resolve this tradeoff differently at different turning rates, producing torque first at low speeds but reducing moment of inertia first at higher speeds. We developed a device that elicits 180° turns repeatedly at controlled speeds. The device constrained fish to perform a turn with a defined radius (a property referred to as maneuverability) while varying the speed of the turn (the agility of the turn). Using this device, we compare the swimming kinematics of bluegill sunfish (*Lepomis macrochirus*) during fast (high agility) and slow (lower agility) turns, while maintaining maneuverability. Across all speeds of turns, lower moment of inertia correlated with faster turns, and minimum moment of intertia tended to coincide with maximum angular velocity. In faster turns, fish began with higher linear momentum, which they then converted to angular momentum. Slower turning fish also took more strokes with their pectoral fins while faster turning fish took higher frequency strokes with their pectoral fins. Therefore we found partial support for our hypothesis: despite substantial variability in the data, for faster turns, fish more often minimized moment of inertia before they maximized torque.

## Introduction

Steady swimming in fishes has been studied extensively, but for many fish species, unsteady swimming comprises a large part of everyday locomotion. Steady swimming, which consists of relatively constant speed forward motion (Webb, 2005), is straightforward to elicit and very repeatable in a flow tunnel (*e.g.*, Hawkins, Di Santo & Tytell, 2025). Unsteady swimming, by contrast, involves maneuvers that change the velocity or the orientation of the body, including forward acceleration or deceleration, left or right turns, or vertical maneuvers (Webb, 2005). These unsteady behaviors are often much more common than steady swimming. Any fish species that lives in a habitat with structure and changing water flow patterns, such as wave-swept coral reefs (*e.g.*, Satterfield, Claverie & Wainwright, 2023) or turbulent rivers with rocky margins (*e.g.*, Tiffan et al., 2009), must maneuver regularly simply to avoid the structures around them. Fishes also maneuver regularly in their interactions with other fishes, in behaviors such as schooling (*e.g.*, Lecheval et al., 2018) and predator–prey interaction (*e.g.*, Rieucau et al., 2014).

For example, trout spend most of their time accelerating, decelerating, and turning rather than swimming steadily (Webb, 1991). Similarly, both zebrafish and tuna, despite their large difference in size, swim unsteadily, turning regularly during normal swimming bouts (Fuiman & Webb, 1988; Howe & Astley, 2020; Thandiackal & Lauder, 2020; Downs et al., 2023). Unsteady swimming becomes particularly important during predator–prey interactions, where both predator and prey accelerate, decelerate, and turn during encounters (Higham, 2007; Hitchcock et al., 2015; Webb, 1983; Webb, 1984a; Webb, 1986). Fish also swim unsteadily during territory defense (*e.g.*, Feldmeth, 1983). Finally, sharks swim unsteadily in social situations such that individuals lower in the hierarchy maneuver to keep out of the way of their superiors (Weihs, Keyes & Stalls, 1981) and will regularly turn facilitated by dramatic body bends (Porter, Roque & Long Jr, 2009; Porter, Roque & Long, 2011).

The best studied unsteady behavior is the C-start, also called an escape response (Domenici & Hale, 2019). Nearly all fish will perform C-starts, which are often responses to attacks by predators (Walker et al., 2005). Since they are a behavior that is critical for survival, they have high angular velocities and occur in short time periods, often occurring in less than one fifth of a second (Domenici & Blake, 1997).

Turning behaviors can be characterized by two main metrics: maneuverability, the ability to turn in a small radius, and agility, the ability to turn at a high angular rate (Fish, 2020). These quantities can vary greatly, both within and across individuals and species. For example, boxfish have rigid bodies and make routine turns in a very tight radius (nearly zero) but at a slow rate (around 200°s^−1^) (Walker, 2000), while giant danios have a more flexible body and perform routine turns more quickly (up to about 3,500°s^−1^) but in a larger radius (typically around 0.3 L) (Howe & Astley, 2020). C-starts can be even faster (up to 5,000°s^−1^) and sometimes with very small radii (0.01*L*) (Howe & Astley, 2020). For any individual, however, lower radius turns (greater maneuverability) tend to be at lower speeds (lower agility) (Bandyopadhyay, 2002; Howe & Astley, 2020).

Since the two metrics are often correlated, it is difficult to identify how fish modulate them. In this study, we developed a new apparatus (inspired by that of Marcoux & Korsmeyer, 2019) that repeatedly elicits 180° turns in a controlled volume (*i.e.,* constant maneuverability) but at different rates (changing agility). This apparatus allowed us to compare the kinematics for slower and faster routine turns.

For these turns, driven mainly by the caudal fin, fish face a tradeoff between torque and moment of inertia. Here, we develop a simple conceptual model to show the tradeoff. Angular acceleration α due to torque at the tail is

(1) \begin{eqnarray*}\mathrm{\alpha }= \frac{{\tau }_{tail}}{I} \end{eqnarray*}

where the torque *τ*_*tail*_ at the tail is

(2) \begin{eqnarray*}{\tau }_{tail}={r}_{tail}{f}_{\mathrm{\perp }}\end{eqnarray*}

and *r*_*tail*_ is the distance from the center of rotation to the center of pressure on the fin and *f*_⊥_ is the force the tail produces that is perpendicular to *r*. For simplicity in this conceptual model, we assume that the force produced by the tail (*f*_⊥_) is constant and unrelated to body bending. Moment of inertia *I* for a body consisting of many segments is

(3) \begin{eqnarray*}I=\sum _{i=1}^{n}{m}_{{}_{i}}{r}_{i}^{2}\end{eqnarray*}

where *m*_*i*_ is the mass of segment *i*, and *r*_*i*_ is the distance from that mass to the center of rotation. For high angular acceleration, fish can increase torque or decrease moment of inertia. Decreasing moment of inertia requires bending the body, which decreases *r*_*tail*_, thus decreasing the torque that can be produced by the tail.

This conceptual model for force production during a turn assumes that the primary turning force is produced by the caudal fin, and that the force is directed roughly perpendicular to the tail. To produce force with the tail, fish must move it laterally, a process that can change the moment of inertia. To develop the conceptual model, we assume that the fish can produce force without moving their tail. This assumption, however, is reasonable when illustrating the tradeoff, because small movements of the tail produce force (Lucas, Lauder & Tytell, 2020). Another limitation of the model is that bluegill sunfish can also turn using mainly their pectoral fins (*e.g.*, Drucker & Lauder, 2001), but these turns tend to be over a much smaller angle than what we studied here, and do not involve much bending of the body.

Dabiri et al. (2020) described one solution for this tradeoff between torque and moment of inertia. They found that both zebrafish and jellyfish initially keep their bodies straight to produce large amounts of torque before bending their bodies to reduce moment of inertia, thus increasing their angular acceleration through the turn. Here, we explored whether this process might change depending on the speed of the turn. Do fish handle these tradeoffs differently when they need to turn quickly rather than when they have time to slowly turn around? We hypothesized that the tradeoffs would be handled differently: that a fast turn is not simply a sped up slow turn. Specifically, in fast turns, we predicted that fish would reduce their moment of inertia earlier in the turn, allowing them to accelerate their rotation earlier.

This prediction follows from Eq. (1): to have a higher angular acceleration, the fish can either increase torque or decrease moment of inertia. Both the torque and moment of inertia depend on *r*, but while torque scales with *r*, moment of inertia scales with *r*^2^ (Eqs. (2) and (3)). Therefore, by bending its body, the fish linearly decreases torque, but quadratically decreases moment of inertia. Since angular acceleration needs to be higher during faster turns, we thus predicted that in fast turns fish would reduce moment of inertia first by bending its body, but in slow turns they would produce torque first by keeping the body straight at the beginning in slow turns, similar to what Dabiri et al. (2020) observed.

We chose to study the bluegill sunfish *Lepomis macrochirus*, a well-studied species (Drucker & Lauder, 2000) thought to be specialized for maneuvering (Webb, 1984a). Maneuvering specialists are thought to have relatively deep bodies (the dorso-ventral height of the body), a narrow caudal peduncle, and a relatively large caudal fin (Webb, 1984a; Webb, 2005). In the field, bluegill typically spend less than about 10% of their time swimming steadily with their caudal fins; instead they more often hold position or perform small turns (Cathcart et al., 2017). For our fast turns, we hoped to see rapid 180° turns in a confined space, a fairly extreme turning behavior, but we also wanted to avoid C-starts, which have a different neural mechanism from routine turns (Domenici & Hale, 2019).

There have not been many fluid dynamic studies of turns driven by body bending. Tytell & Lauder (2008) studied the hydrodynamics of C-starts, and Thandiackal & Lauder (2020) studied routine turns in zebrafish. Both found that the turns generate a jet of fluid, entrained by the body as it bends, which is then shed off the tail as the fish kicks out of the turn. We assumed that the hydrodynamics of the routine turns studied here should be relatively similar. Importantly, both of these studies showed that a substantial proportion of the force is produced by the caudal body and fin, and that the jet is roughly perpendicular to the body and fin, a pattern that supports our conceptual model of the tradeoffs between torque and moment of inertia.

Therefore, we examined the mechanism proposed by Dabiri et al. (2020) to test whether it holds for turns at different speeds. Inspired by the system built by Marcoux & Korsmeyer (2019), we developed a system that allows us to repeatably elicit 180° turning behavior at controlled speeds. The system consists of a flow-through chamber attached to a movable platform that is mounted on top of a flow tank. Fish placed in the chamber reliably turn as the chamber changes direction. Portions of this text were previously published as part of a preprint (Clark & Tytell, 2025).

Using this system, we worked with bluegill sunfish (*Lepomis macrochirus*) to examine the kinematics of slow and fast 180° turns. We quantified linear and angular momentum of the body during the turn to examine how fish exchange linear and angular momentum, and whether this exchange might differ depending on speed. Overall, we aimed to answer the question: are fast turns simply sped up slow turns, or are there distinct kinematic patterns that differentiate them?

## Materials & Methods

### Animals

We collected six bluegill (*Lepomis macrochirus*) from either White Pond or Walden Pond in Concord, MA. We aimed to collect adults around 15 cm in total length, because that size individual fits best within the turning apparatus. We used a seine net along the shores of these ponds to collect the fish before transferring them to an aquaculture facility where they were kept in 10 gallon tanks at a controlled temperature of 20 °C until used for experiments. We collected the fish with scientific collection permits approved by the Massachusetts Division of Fisheries and Wildlife (permit numbers 139.24SCF, 146.23SCF, 090.22SCF, and 174.20SCF).

No animals suffered any adverse effects from these behavioral experiments. All animals survived the procedures and have continued to be maintained at Tufts University.

All experimental procedures and animal handling protocols were approved by the Tufts University Institutional Animal Care and Use Committee (protocols M2018-103, M2021-99, and M2024-57).

### Setup

**Figure 1:**
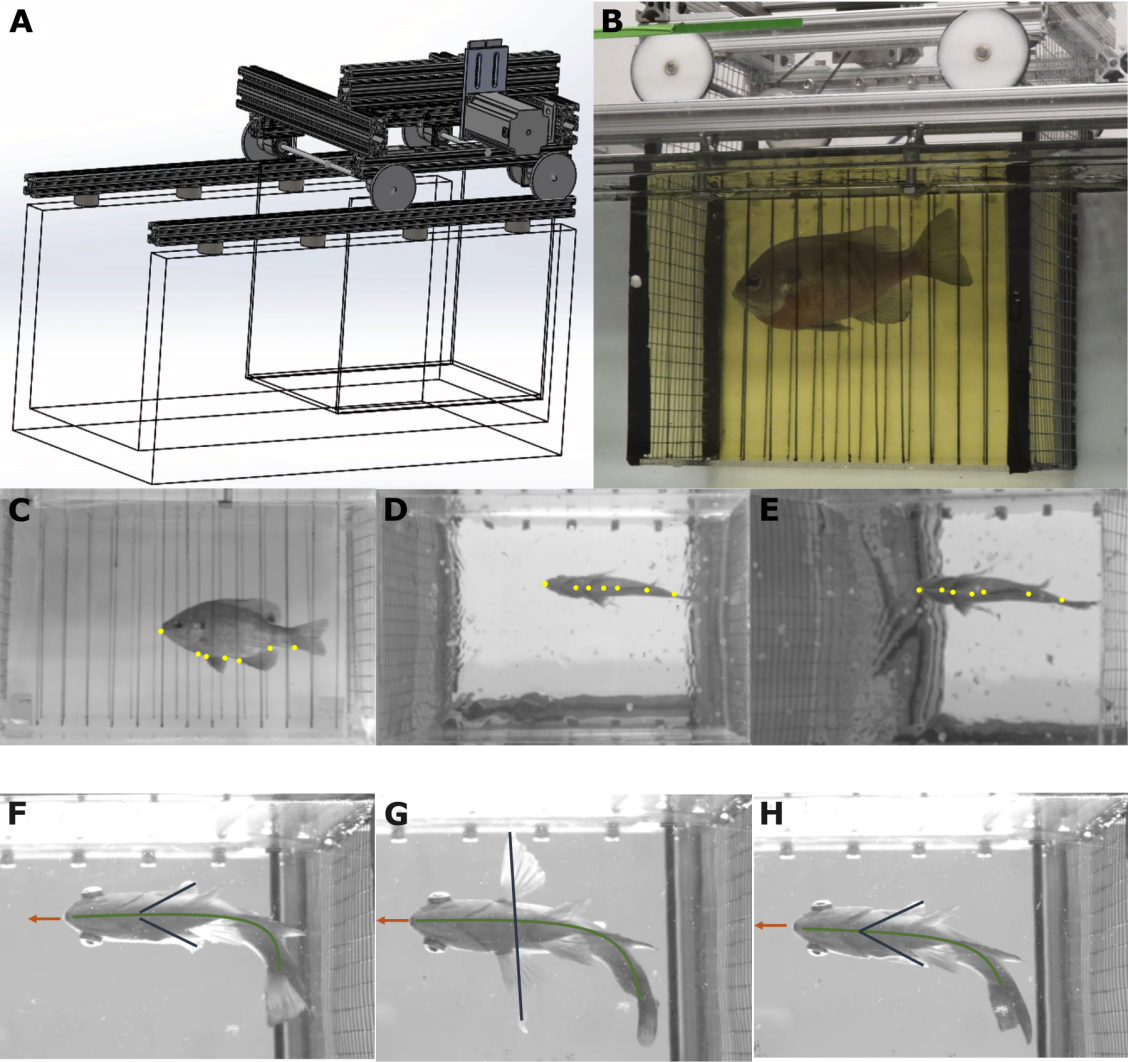
The programmable car elicits repeatable turns. (A) A 3D design of the system (available in Supplementary Material). (B) A bluegill swimming in the chamber. (C, D, E) Sample images from the orthogonal lateral camera (C), orthogonal ventral camera (D), and the off axis ventral camera (E). The yellow points represent digitized points along the body. Light gray lines show the skeleton used for training (the snout to all other points to form a shallow tree). (F, G, H) Three consecutive turns from the ventral view, showing similar kinematics.

We used an oscillatory device (referred to as a “car”) to repeatably induce turning behaviors (Figs. 1A, 1B). We mounted a Teknic ClearPath-SDSK servo motor (part number CPM-SDSK-2310S-EQN) to an 80/20 chassis with an attached acrylic flow-through chamber that is 20.3 cm wide by 30 cm long and 30.5 cm tall. When on top of the flow tank, the water level sits about 19.3 cm above the bottom of the flow through chamber. This results in a functional volume that is 20.3 cm wide, 30 cm long, and 19.3 cm tall. This device drove forwards and backwards at programmable rates controlled by an Arduino Uno. The flow-through chamber had metal grates on either end to keep the fish inside and translucent plastic sheets taped on either side with vertical lines drawn on them to provide a visual stimulus for the fish. The wheels of the car ran in a metal track. Since the fish is contained by the grates of the car, when the car reverses direction, the fish must turn at roughly the same rate.

For all trials, we kept the water still while the car moved along the track and ran the car at either a low speed (around 1 body lengths, *L*, per second at peak speed) or a high speed (either 2.5 or 3.5 *L*/s at peak speed) to elicit slow and fast turns respectively. Fish were gently encouraged to remain centered in the flow-through chamber with a wooden dowel. We defined the start of each turn as the first observable movement of the snout in the direction of the turn, and the end as when the fish’s snout was oriented in the opposite direction (an approximately 180° turn). These turns were repeatable (see Figs. 1F–1H for an example) and their consistency allowed us to collect multiple trials with similar performance.

Before the experiment, a fish was transferred into the chamber and allowed to acclimate for 5 to 30 min. We then began the car movement at a low speed to allow fish to adapt to the car. In general, for each trial, fish adapted to the movement in the car within 1 or 2 car cycles, and stopped showing signs of stress such as erratic movements, attempted escapes, or rapid gilling. One individual never fully acclimated to the chamber, and was therefore excluded from the data set. We ran all our experiments over the course of a few hours, but recorded each series of slow and fast turns in around 10 min or less. We gathered the data in blocks of 16 consecutive turns at the same speed, but randomized the order of speeds.

### Flow disturbance induced by the apparatus

To quantify the disturbance induced by the movement of the car, we used standard particle image velocity (Tytell, 2011). A horizontal light sheet was projected through the middle of the car (1,000 mW, 532 nm, Opus MPC 600, Laser Quantum, San Jose, CA, USA). Titanium dioxide particles (Vetosint 1141 white, Degussa Co, Piscataway, NJ, USA) were added to the tank and the light sheet was filmed at 400 frames per second (fps) with an exposure duration of 2,000 µs. The PIV algorithm was performed with PIVLab (v3.09; Thielicke, W., http://www.pivlab.de/) in Matlab (R2024b, MathWorks, Natick, MA, USA) and calibrated spatially with a ruler. Across all speeds, the flow in the center of the car was 6.6% of the car speed, in the opposite direction as the car, with a turbulence intensity of about 10% of the car speed. The highest induced flow was at an intermediate car speed, in which the flow was 9.8% of the car speed.

### Filming and digitizing

To film the kinematics, we used two Phantom Miro M340 high-speed cameras and one Phantom Miro M120 high-speed camera. We positioned two cameras orthogonal to each other: one directly below the working section of the tank and one directly lateral to the working section of the tank, with one positioned ventrally and at an angle to the rear to the other cameras. We filmed all videos at 60 fps. We calibrated videos by using a ChArUco board (Garrido-Jurado et al., 2014) and the aniposelib package from the Anipose toolkit (Karashchuk et al., 2021).

We used SLEAP (Pereira et al., 2022) to digitize seven points along the midline on the bottom of the fish: the snout, a point in line with the posterior portion of the gills, a point between the pelvic fin insertions, a point halfway between the pelvic fin insertions and the anal fin insertion, the anal fin insertion, a point halfway between the anal fin insertion and the caudal peduncle, and the peduncle itself (Figs. 1C–1E). We wrote custom code to use a ChArUco board to calibrate the filming space using aniposelib (Karashchuk et al., 2021) and triangulated all digitized points from SLEAP into 3D coordinates in centimeters. We then rotated all points such that they conformed to axes defined by the flow-through section of the apparatus, where the positive *x* axis is forward, the positive *y* axis is to the fish’s left, and the positive *z* axis is up. Finally, we used the projections of the 3D points into the *x*, *y* plane for all analyses, since those were the primary axes in which the turns took place, but corrected for parallax introduced if the fish was at different vertical locations.

We smoothed the *x* and *y* locations of all digitized points individually using a 9th order low-pass Butterworth filter with a cutoff frequency of 8 Hz for fast turns and 4 Hz for slow turns. We then used a cubic smoothing spline to spline the points along the body, interpolating locations that are evenly spaced along the body.

### Snout and tail angle and angular velocity

We estimated snout angle by first drawing a line from the center of the pelvic fin insertions to the snout, and tail angle similarly with caudal peduncle and the point halfway between the base of the anal fin and the peduncle. The angle between this vector and the horizontal *x* axis (*θ*) is then $\theta =\mathrm{atan}2 \left( {y}_{1}-{y}_{2},{x}_{1}-{x}_{2} \right) $, where *y*_1_ and *x*_1_ are the y and x positions of the snout and *y*_2_ and *x*_2_ are the y and x positions of the pelvic point, respectively. We used the same methods to calculate the tail angle, but using the peduncle point instead of the snout point and the base of the anal fin point instead of the pelvic point.

We smoothed the angles using a low-pass Butterworth filter with a threshold of 4 Hz before numerically differentiating the angle with respect to time by estimating the central difference between points (Smith, 1986). Positive angular velocity values denote movement in the direction of the turn while negative angular velocity values denote movement in the direction opposite the turn.

### Moment of inertia

**Figure 2:**
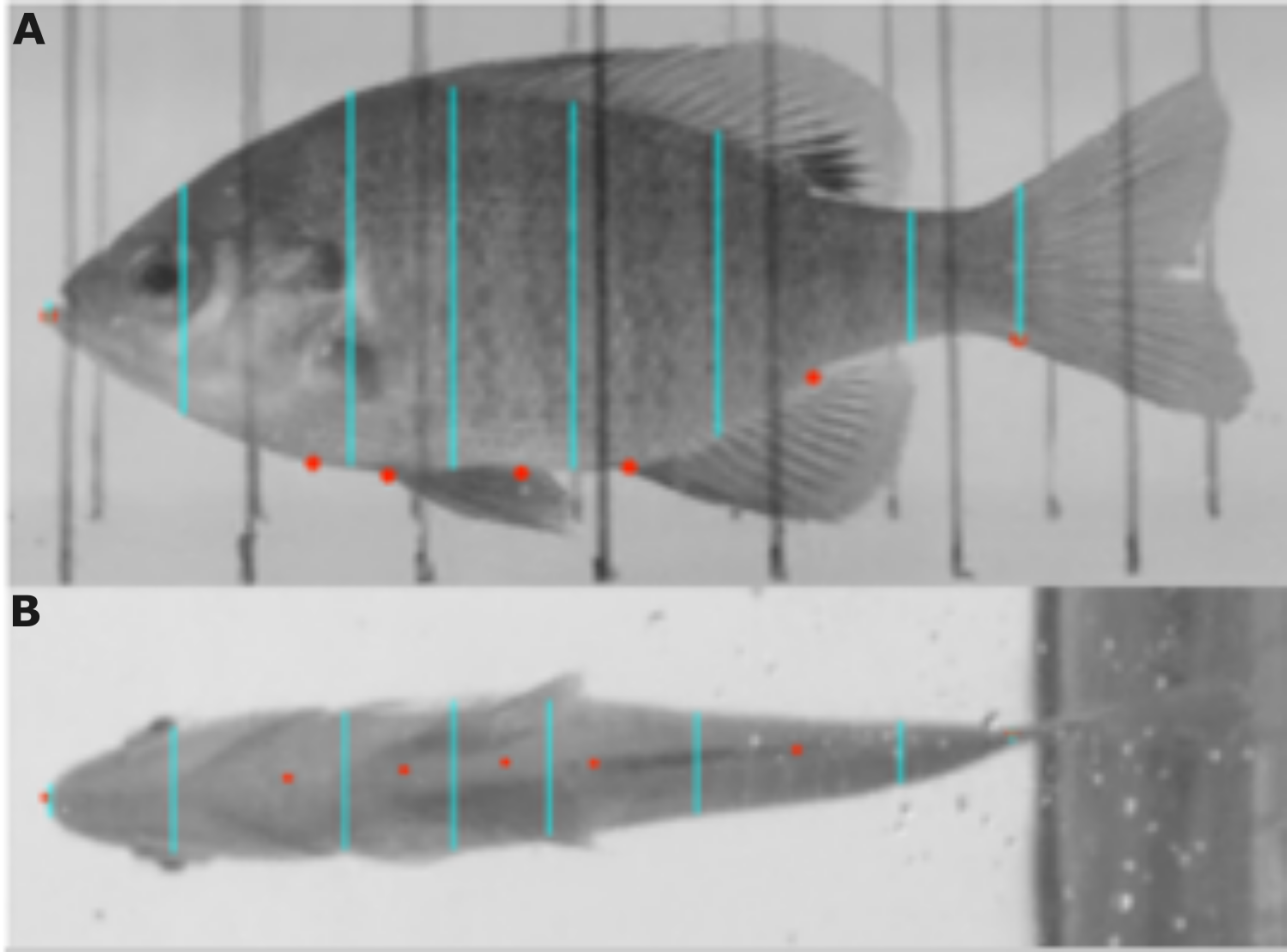
Mass distribution was estimated using images of each bluegill. (A, B) Points were added to their respective locations along the length of the body (red dots). Lines were then drawn from vertical edge to vertical edge of the fish in the lateral view (A) and lateral edge to lateral edge of the fish in the ventral view (B) (light blue lines). FIJI was then used to measure each of the lines and the distance between them in the ventral view. The known length of the fish was used to set the scale.

To estimate the moment of inertia, we must first estimate the center of mass for the flexible body as it bends. This requires an estimate of the mass of each segment of the body. An accurate way (Fath et al., 2023; Roche, Tytell & Domenici, 2023) to estimate these masses is to approximate the volume of a segment as a truncated elliptical cone and assume that the body density is constant. We used FIJI (Schindelin et al., 2012) to measure height *h*, width *w*, and length Δ*l* of each segment of the fish (Fig. 2). We drew each segment line halfway between two adjacent points down the length of the body, with the exception of the snout and peduncle point where we drew one segment line at the point. For each point along the body, we used the width and height of the body segment line directly anterior to the point (*w*_*i*_ and *h*_*i*_) and the width and height of the body segment line directly posterior to the point (*w*_*i*+1_ and *h*_*i*+1_).

The volume *V*_*i*_ of each segment is thus

(4) \begin{eqnarray*}{V}_{i}=\pi \Delta l \left( {w}_{i}{h}_{i}+ \frac{1}{2} \Delta w{h}_{i}+ \frac{1}{2} \Delta h{w}_{i}+ \frac{1}{3} \Delta w\Delta h \right) \end{eqnarray*}

where Δ*l* is the distance between segments, Δ*w* = *w*_*i*+1_ − *w*_*i*_ and Δ*h* = *h*_*i*+1_ − *h*_*i*_, and the mass of each segment is then *m*_*i*_ = *ρV*_*i*_ (Roche, Tytell & Domenici, 2023).

The moment of inertia Eq. (3) was then estimated, assuming each segment was a point mass located at its centroid and that the center of rotation was the whole body center of mass (*X*_*M*_, *Y*_*M*_), which we estimated as

(5) \begin{eqnarray*}{X}_{M}= \frac{\sum {x}_{i}{m}_{i}}{{m}_{t}}  \mathrm{and} {Y}_{M}= \frac{\sum {y}_{i}{m}_{i}}{{m}_{t}} \end{eqnarray*}

where *m*_*i*_ is the mass of each segment, *m*_*t*_ is the total mass of the fish, and *x*_*i*_ and *y*_*i*_ are the x and y positions of the center of each segment respectively. Instantaneous moment of inertia was normalized by dividing by the moment of inertia for each individual when its body was straight.

### Torque

We estimated the whole body torque of the fish based on of the sum of the torques of the segments. In a non-rigid body, torque is the time derivative of moment of inertia of each body segment (*I*_*i*_) multiplied by its corresponding angular velocity (*ω*_*i*_)

(6) \begin{eqnarray*}\tau =\sum \frac{d}{dt} \left( {I}_{i}{\omega }_{i} \right) \end{eqnarray*}

where the moment of inertia *I*_*i*_ for each segment is equal to the segment’s mass multiplied by the square of its distance to the center of rotation (*r*_*i*_). Evaluating the derivative gives an estimate of the whole body torque of the fish

(7) \begin{eqnarray*}\tau =\sum {m}_{i} \left( 2{r}_{i}\omega {\dot {r}}_{i}+{r}_{i}^{2}{\dot {\omega }}_{i} \right) .\end{eqnarray*}

### Pectoral fins

We manually digitized the start of each pectoral fin stroke that occurred during the turn. Working with only ventral orthogonal videos, we identified the frames in which each pectoral fin was maximally extended away from the body, immediately before the fish pulled it back towards the body to take a stroke. We digitized the tips of each pectoral fin when it was extended in a frame and the bases of both pectoral fins in all frames in which either fin was extended. In many instances, bluegill seem to be taking “backing” strokes in which they pushed their pectoral fins far towards their snout to produce a backwards force on that side. For our analyses, we defined a backing stroke to be any pectoral fin stroke toward the anterior body with an amplitude greater than 90° at maximal extension.

### Momentum

For turning, fish may be able to convert linear momentum into angular momentum. We therefore estimated the linear momentum *p*_*l*_ of the fish during turning as

(8) \begin{eqnarray*}{p}_{l}={m}_{t}{v}_{l}\end{eqnarray*}

where linear velocity *v*_*l*_ is the central difference of the position of the center of mass Eq. (5) differentiated with respect to time and *m*_*t*_ is the total mass of each fish. Linear momentum has units of kgms^−1^; to account for size differences among individuals, we therefore compare a normalized momentum ${p}_{l}^{\mathrm{\ast }}$ by dividing by the fish’s mass and its body length, ${p}_{l}^{\mathrm{\ast }}={p}_{l}/ \left( {m}_{t}L \right) $.

We then estimated angular momentum *p*_*θ*_ as

(9) \begin{eqnarray*}{p}_{\theta }=I{v}_{\theta }\end{eqnarray*}

*I* is the moment of inertia we estimated using Eq. (3) and *v*_*θ*_ is the angular velocity of the fish’s snout as estimated above. Similarly, since angular momentum has units of kgm^2^degs^−1^, we compare normalized angular momentum ${p}_{\theta }^{\mathrm{\ast }}$ by dividing by mass and body length squared, ${p}_{\theta }^{\mathrm{\ast }}={p}_{\theta }/ \left( {m}_{t}{L}^{2} \right) $.

For both linear and angular momentum, we estimated the pre-turn momentum, defined as the mean of the momentum in the two frames leading up to the turn, and the mean momentum during each third of the turn, dividing the turn into three parts according to the total time of each turn.

### Statistics

For all analysis and visualization, we used R version 4.3.3 with Rstudio version 2023.12.1.402 and the following packages: tidyverse version 2.0.0 (Wickham et al., 2019), lme4 version 1.1.35.1 (Bates et al., 2015), car version 3.1.2 (Fox & Weisberg, 2019), ggspatial version 1.1.9 (Dunnington, Thorne & Hernangómez, 2023), performance version 0.14.0 (Lüdecke et al., 2021), ggdist version 3.3.2 (Kay, 2024), and beeswarm version 0.4.0 (Eklund & Trimble, 2021).

Our primary experimental unit was a single turning behavior. We had five individuals animals in the data set, each of which contributed at least 15 turns at each speed. To account for repeated measurements from the same individual, we use mixed model statistics including individual as a random effect.

Criteria established a priori for excluding a behavior were when the fish touched the side or back of the chamber or were pitched up or down more than about 30°, or, on rare occasions, when they displayed signs of stress as described above. We excluded at most one turn per individual, for a total of three behaviors that were excluded from the data set.

We conducted a power analysis using ‘mixedpower’ (Kumle, Võ & Draschkow, 2021), a simulation-based approach for power analyses with mixed models. We tested the power to detect differences in the timing of maximum torque and minimum moment of inertia, the key comparison for our hypothesis. With five individuals, we have a 98% power to detect differences of the magnitude we observed at an α level of 0.01, based on 1,000 simulated analyses.

When we used pairwise comparisons to look at the effect of the “slow” and “fast” speed groups, or to look for significant differences between changes in momentum and turn portion, we used the Anova function from the car package and the lmer function from the lme4 package to run an ANOVA on a linear mixed-effects model with individual as a random effect. Model assumptions were checked visually using the check_model function from the performance package. When we present an exponential fit for fin beat frequency, we used the lmer function to fit a 1/*x* curve.

To see if the time differences between time of maximum torque and time of minimum moment of inertia were significant, we used emmeans (Lenth et al., 2025) to compare each group to 0 with individual as a random effect. We also fit a regression model of maximum angular velocity to the time difference between maximum torque and minimum moment of inertia using lmer, with individual as a random effect.

All means are provided plus or minus one standard deviation, unless otherwise noted.

## Results

We collected turning behavior from five individuals (length 15.6  ±  3.2 cm; mean   ±   sd), and each individual contributed at least 15 slow and 15 fast turns to the data set. See Supplemental Movies (Movie 1 and Movie 2) for examples of turns.

### Angular velocity was higher when the car reversed faster

**Figure 3:**
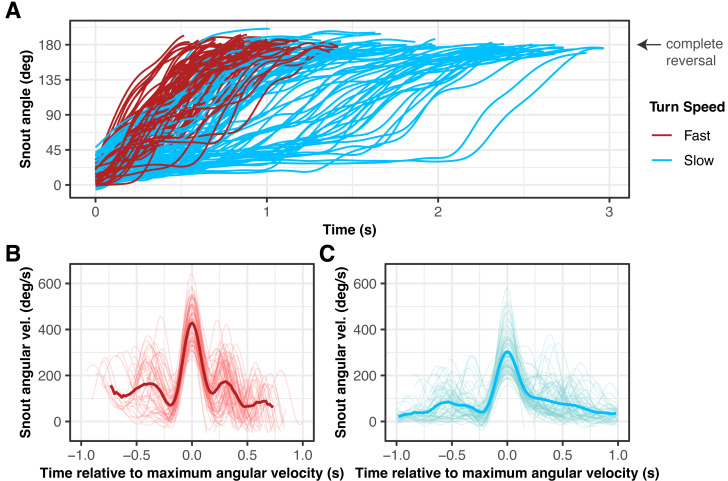
Angular velocities were higher when the car reversed faster. (A) Snout angle relative to time for slow turns in blue and fast turns in red. (B, C) Angular velocity with fast turns in red (B) and slow turns in blue (C).

During fast car reversals (which we will call “fast turns”), fish turned 180° in less time than they did during slow car reversals (“slow turns”), resulting in higher angular velocities (Fig. 3). For each turn, we took the mean and maximum angular velocity over the duration of the turn. Then, we examined the means of these values across all turns. Both the mean (across trials) of the mean (within a trial) and the mean maximum snout angular velocity were significantly higher in fast turns (mean = 181 ± 41°s^−1^; max = 428 ± 84°s^−1^) relative to slow turns (mean = 96 ± 35°s^−1^, max = 303 ± 89°s^−1^; *p* < 0.001 for both mean and maximum comparing fast to slow turns).

### Changes in snout angular velocity were driven by anterior bending

**Figure 4:**
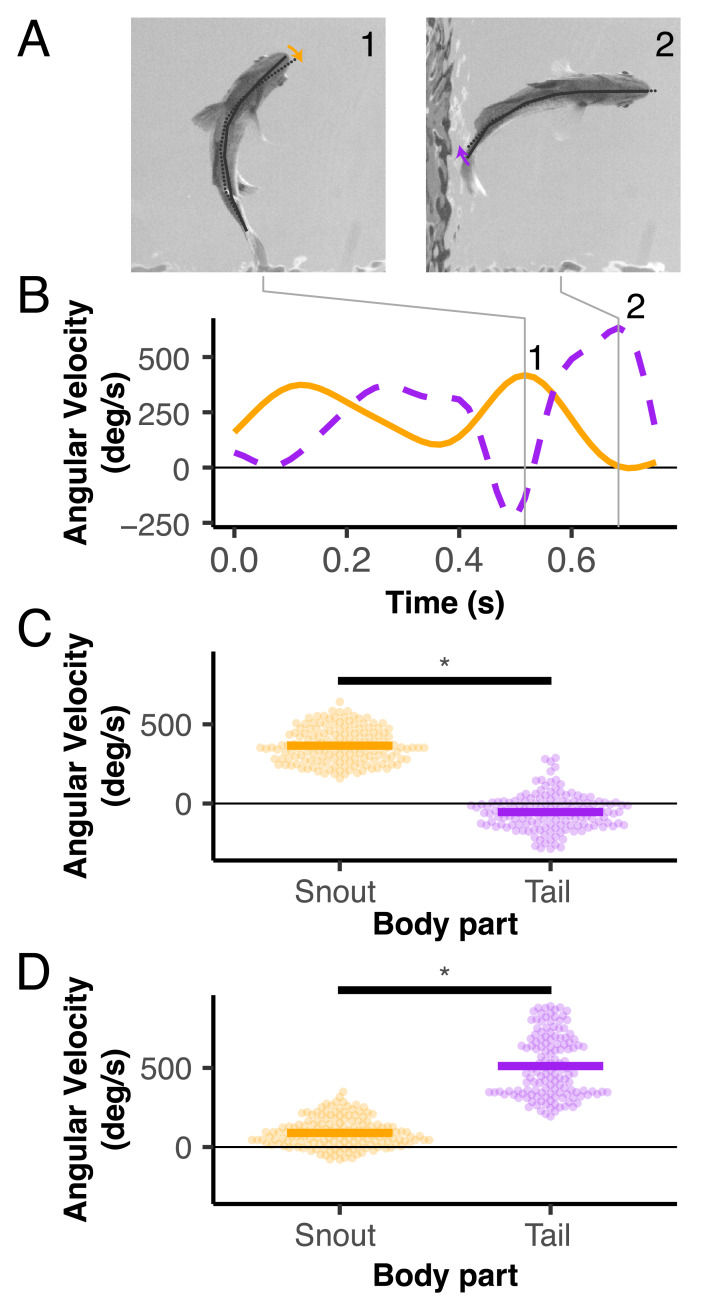
Snout and tail angular velocities across both slow and fast turns. (A, B) Representative frames and traces of one trial’s angular velocities over time for the snout (orange) and tail (purple). The representative turn is a fast turn. The gray lines represent the time of maximum angular velocity for the snout (frame 1) and tail (frame 2). (B) Snout and tail angular velocities for each trial at the point where snout angular velocity is maximized (gray line 1 in A). Each point denotes the value for one trial. (C) Snout and tail angular velocities at the point where tail angular velocity is maximized (gray line 2 in A). Asterisks (*) denote significant differences between groups (*p* < 0.05).

When the snout was moving at its maximum angular velocity, the tail was moving at a significantly lower angular velocity (*p* < 0.001) and was close to 0 (Fig. 4B). The reverse was also true: when the tail was moving at its maximum angular velocity, the snout was moving at a significantly lower angular velocity (*p* < 0.001) that was close to 0 (Fig. 4C).

Additionally, for all turns, fish asymmetrically beat their caudal fin and also simultaneously beat the median fins to that same side as the caudal fin. In about 1/3 of fast turns, fish bent and held their median fins to the side of the turn before beginning to beat their tails.

### The minimum moment of inertia was similar for slow and fast turns

**Figure 5:**
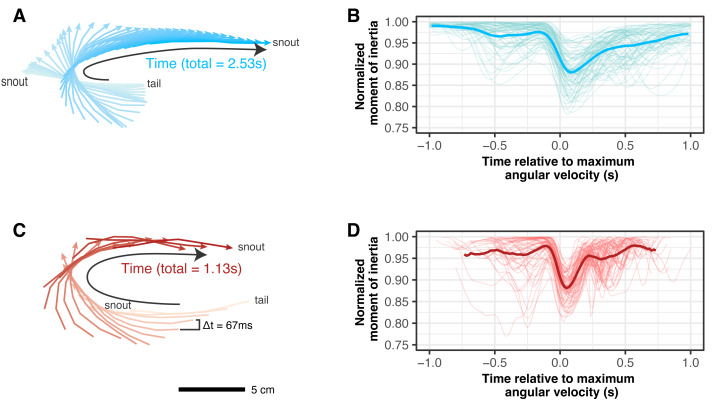
Fish bent their bodies to a similar degree in slow and fast turns. (A, C) Body traces of midlines for one individual for one slow (A) and one fast (C) turn. The snout is labeled with an arrow head. The darkness of the line color in (A) and (C) corresponds to the time course of the turn with lighter colors representing the beginning. Time between frames is the same for both sequences. (B, D) Normalized moment of inertia for slow (B) and fast (D) turns. Time is centered on the time of maximum angular velocity.

Whether fish turned slowly or quickly, they bent their bodies by about the same amount, decreasing their moment of inertia to the same mean value, despite different turning rates (Fig. 5). The mean value of the minimum normalized moment of inertia across the duration of a slow turn was 0.9  ±  0.04 and for fast turns was 0.9 ± 0.03 (*p* = 0.906).

### Lower moments of inertia resulted in higher angular velocities within each turn speed

**Figure 6:**
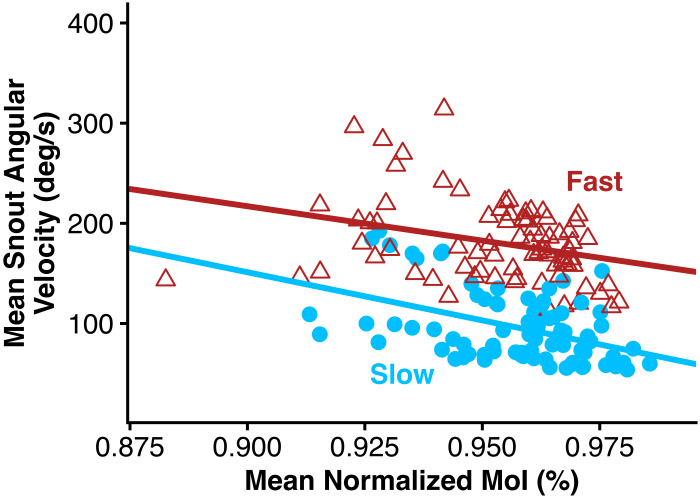
Within each turn speed, bluegill that bent their bodies more generated higher angular velocities. Mean moment of inertia per trial, normalized by the maximum moment of inertia for that trial, plotted against mean snout angular velocity of that trial. Data is shown for both slow (in blue) and fast turns (in red). Linear regressions for slow (blue) and fast (red) turns are shown.

Although the minimum moment of inertia did not differ significantly across speed groups, there was substantial variance within each group. Comparing within each turn speed, fish that bent their bodies more or held the bend longer over the course of the entire turn (resulting in a lower mean moment of inertia) turned with higher mean angular velocities (Fig. 6). Fitting with a linear mixed effects regression with individual as a random effect yielded a significant negative slope for both slow (*p* < 0.001) and fast (*p* < 0.001) turns. The slopes were the same for both types of turns (*p* = 0.408).

**Figure 7:**
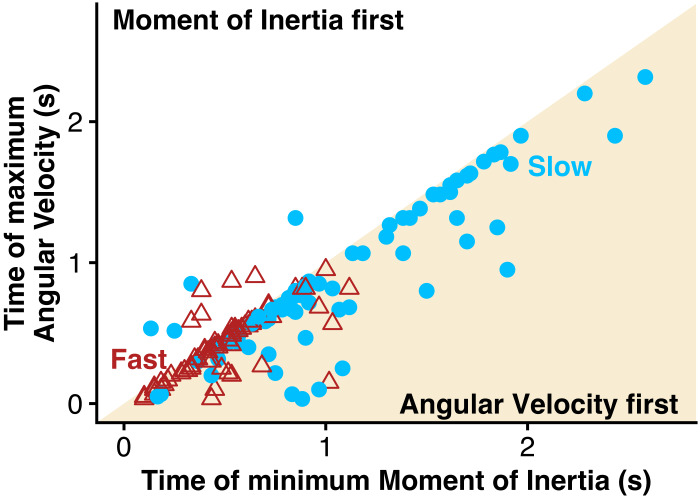
Bluegill usually maximized angular velocity before minimizing moment of inertia. The time at which moment of inertia was minimized during a turn is plotted against the time at which angular velocity was maximized during that turn for both slow (blue) and fast (red) turns. The yellow shaded region is where the fish maximized its angular velocity before minimizing its moment of inertia, while the unshaded region is where the fish minimized its moment of inertia before maximizing its angular velocity.

Comparing across speed groups, the mean angular velocity was significantly higher in fast turns (*p* < 0.001; Fig. 3) even though the mean moment of inertia did not differ significantly between slow and fast turns (0.95  ±  0.02 for both, *p* = 0.114),

Fish almost always reached their maximum angular velocity just before they fully minimized their moment of inertia (points in the bottom right shaded region in Fig. 7), regardless of turn speed. Both slow and fast turns followed this pattern with few exceptions (only nine out of 139 trials).

### Fast turns traded off linear and angular momentum

**Figure 8:**
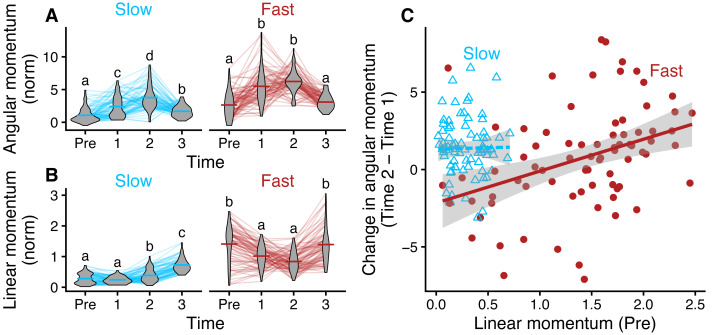
Bluegill converted linear to angular momentum in fast turns but not slow ones. (A) Angular momentum for slow (blue) and fast (red) turns. (B) Linear momentum for slow and fast turns. Groups that are significantly different from one another are labeled with different letters (*p* < 0.05). Letters are unique within linear momentum and angular momentum separately and within Slow and Fast. “Pre” refers to the momentum in the 30 ms before the first movement of the turn. Turns were divided in three parts temporally, where the end of the turn was when the snout completed the turn through 180°. (C) Relationship between the initial linear momentum and the increase in angular momentum from time 1 to time 2 for slow turns (blue) and fast turns (red). Solid lines indicate significant relationships (*p* < 0.05).

Angular momentum showed a similar pattern across the turns (Fig. 8A) while linear momentum differed between the two types of turns (Figs. 8C, 8D). Pre-turning linear momentum was significantly higher in fast turns than it was in slow turns (*p* < 0.001). For fast turns, linear momentum then significantly *decreased* in both the first and second third of the turn (*p* < 0.001 in both cases). However, for slow turns, it did not change significantly in the first third of the turn (*p* = 0.141) and significantly *increased* in the second and third parts of the turn compared to pre turning phase (*p* < 0.001). In other words, in fast turns, bluegill initially started with higher linear momentum, which decreased during the entire turn, corresponding to an increase in angular momentum (Fig. 8). In slow turns, bluegill initially started with low linear momentum (Fig. 8), increased angular momentum in the first half of the turn while maintaining linear momentum, then increased linear momentum in the second half of the turn as angular momentum decreased (Fig. 8).

For fast turns only, turns that had higher initial linear momentum also had larger increases in angular momentum (Fig. 8C; *p* < 0.001). Slow turns did not have a significant relationship between the two (*p* = 0.851).

### Bluegill used their pectoral fins differently during fast turns

**Figure 9:**
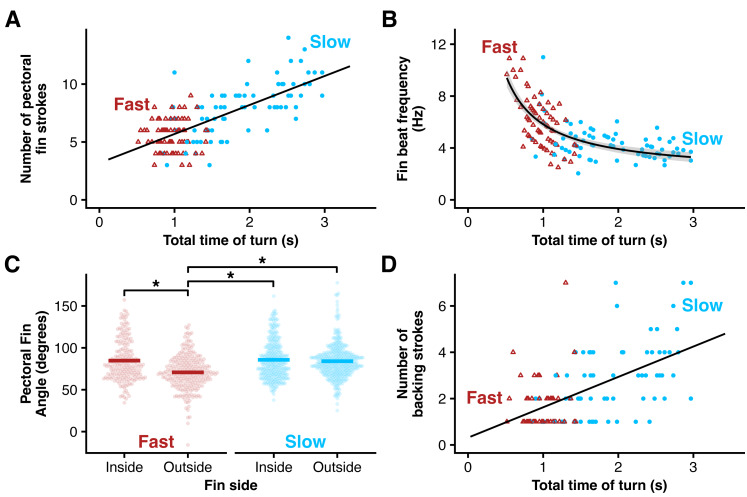
Bluegill used their pectoral fins differently at different speeds. Pectoral fin data for slow (blue circles) and fast (red triangles) turns. (A) Total number of pectoral fin beats increases for longer turns. The black line is the linear fit. (B) Fin beat frequency decreased for longer turns with a 1/x model shown by the black line. (C) Fin amplitude for the inside and outside fins in slow and fast turns. (D) Number of backing strokes increased for longer turns. The black line shows the linear fit. Asterisks denote differences between groups (*p* < 0.05).

Bluegill took more strokes with their pectoral fins in slower turns (based on a significant positive slope on the regression: *p* < 0.001) (Fig. 9A), but beat their pectoral fins at a higher mean frequency in fast turns (based on a significant inverse relationship on the regression: *p* < 0.001) (Fig. 9B). During slow turns, fish beat both their inside and outside pectoral fins at a similar amplitude (*p* = 0.378), but during fast turns, fish beat their inside fin at a higher amplitude than their outside fin (*p* < 0.001) (Fig. 9C). The inside fin amplitudes did not differ between slow and fast turns (*p* = 0.643) (Fig. 9C). Finally, bluegill took fewer backing strokes during fast turns (*p* < 0.001) (Fig. 9D). All individuals used their pectoral fins in all turns.

### Timing of maximum torque and minimum moment of inertia differed based on turn speed

**Figure 10:**
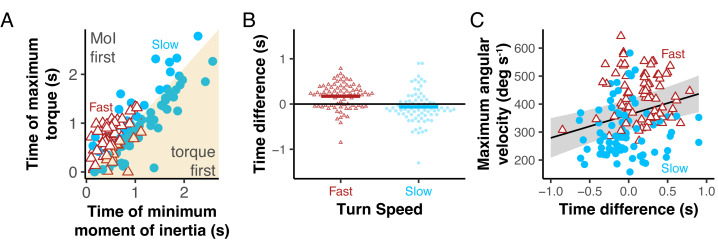
Slow and fast turns differed in their timing of maximum torque and minimum moment of inertia. (A) The time at which moment of inertia was minimized during a turn is plotted against the time at which torque was maximized during that turn for both slow (blue) and fast (red) turns. The yellow shaded region is where the fish maximized its torque before minimizing its moment of inertia. (B) The difference between the time that the bluegill maximized their torque and minimized their moment of inertia is show for both fast (red) and slow (blue) turns. (C) Turns with a positive time difference (MoI minimized before torque) had higher maximum angular velocities.

The timing of the estimated maximum torque relative to the minimum moment of inertia was different for slow and fast turns (Fig. 10). In slow turns, fish maximized torque before minimizing moment of inertia in 65% of turns. In fast turns, this only happened in 31% of the turns. For slow turns, fish minimized their moment of inertia a mean of 0.06  ±  0.3s *after* maximizing torque, which was significantly different than fast turns, where fish minimized moment of inertia 0.2  ±  0.3s *before* maximizing torque (*p* < 0.001) (Fig. 10B). For slow-turning fish, the difference between the time at which they maximized torque and minimized moment of inertia (Fig. 10B) was negative (torque first), but not significantly different from zero (*p* = 0.093), whereas for fast turning fish, the difference was significantly positive (torque second) (*p* < 0.001). Positive time differences (MoI first) correlated significantly with higher maximum angular velocities (Fig. 10C; *p* < 0.0001, mixed regression model with individual as a random intercept).

## Discussion

**Figure 11:**
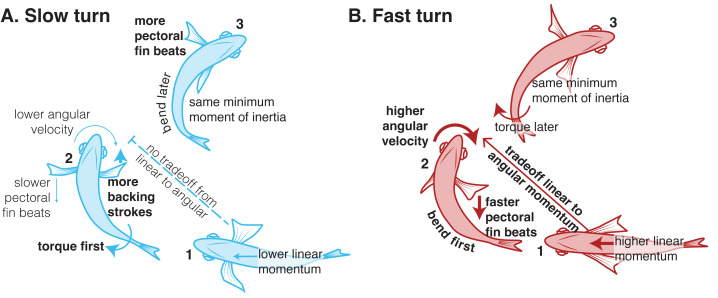
Summary of the similarities and differences in slow and fast turns, showing three time points within each type of turn (numbered 1 to 3).

Figure 11 shows a summary of the similarities and differences in slow and fast turns. Most of the kinematics are quite similar. Even though the fish performed the fast turns with about twice the angular velocity as slow turns, they bent their bodies to about the same degree in each case, reaching the same minimum moment of inertia (Fig. 5). For both types of turns, they began turning with an anterior body movement (Fig. 4), “planting” the tail as the snout moved laterally. In both types of turns, what differentiated faster turns from slower ones was the duration of the low moment of inertia. Faster turns in either group maintained the low moment of inertia for longer, resulting in a lower mean moment of inertia (Fig. 6).

But with the same mean moment of inertia, the fast turn group had higher angular velocity (compare blue and red points in Fig. 6). How is that possible? First, bluegill traded off linear and angular momentum differently in slow and fast turns. Fast turns began with relatively high linear momentum, which the fish were able to convert to angular momentum during the turn (Fig. 8C). This conversion seems to be driven both by body bending, particularly in the anterior body (Fig. 3) early in the turn (Fig. 5), but also by the pectoral fins. The inner pectoral fin beats faster and at a higher amplitude during fast turns compared to slow turns, likely producing higher forces (Fig. 9), a pattern that should aid in the conversion of linear to angular momentum. During slow turns, they had lower linear momentum, which meant that they needed to provide power to increase both angular momentum during the turn and linear momentum as they came out of it (Figs. 8A, 8B). Different from fast turns, slow turns had no significant differences between pectoral fin beat amplitude on the inside and outside of the turn (Fig. 9C), suggesting that the increase in angular momentum in the slow turn is produced primarily by body bending, perhaps with the pectoral fins serving more for stability.

The programmable car was effective at eliciting repeatable turns, but it is clearly different from a fish’s natural habitat. The visual and flow stimulus from the car triggers a turn, and the fish did not interact physically with the car in any way. Moreover, our PIV results demonstrate that the flow inside of the car is not dramatically altered by its movement. Thus, we argue that, if a fish were to perform a 180° turn without the car, it would do it in a similar way. More importantly, they would face the tradeoff between torque and moment of inertia whether they turn in the car or spontaneously.

The turns studied here are relatively extreme, but we suggest that they are nevertheless relevant to the ecology of bluegill sunfish. Turns through any angle face the same tradeoff between tail torque and moment of inertia as we described here, which will be relevant in the wild, where bluegill rarely perform 180° turns (Cathcart et al., 2017). They often turn with a small radius of curvature, around 1BL (Cathcart et al., 2017), which is similar to the turns we studied here. Fast and large turns require a lot of energy (Cathcart et al., 2017; Ellerby et al., 2018), which likely explains why they were not observed very often. Behaviorally, however, such turns may be very important, as they help the fish to avoid obstacles, attack prey, or avoid predators.

Our results do not indicate how muscle is recruited during these turns. Slow steady swimming is driven exclusively by red muscle (Jayne & Lauder, 1993), while turning recruits some white muscle (Jimenez & Brainerd, 2021), and fast starts recruit even more (Jimenez & Brainerd, 2021). It may be that even relatively slow turning behavior requires the higher forces produced by white muscle (Shadwick & Gemballa, 2006) due to the high body curvature. This hypothesis could be tested in future electromyography studies, which should compare red and white muscle recruitment during behaviors with high curvature, even if the behaviors are relatively slow.

### Fast turns trade off torque and inertia differently from slow turns

We suggest that the main difference between fast and slow turns, however, is the tradeoff between torque and moment of inertia. We hypothesized that bluegill would change the timing of maximum torque and minimum moment of inertia for turns at different rates. Due to how torque scales with *r* (Eq. 2), while moment of inertia scales inversely with *r*^2^ (Eq. 3), a reduction in *r* decreases moment of inertia more than torque. Therefore, in fast turns, we predicted that bluegill would prioritize minimizing moment of inertia early to maximize angular acceleration at the start.

Our results partially supported this hypothesis. Bluegill minimized moment of inertia significantly earlier in fast turns than in slow turns (Fig. 10). In most fast turns (70%), the fish minimized moment of inertia before they maximized torque, where in slow turns, they tended to maximize torque first. For slower turns, they used a similar pattern to what Dabiri et al. (2020) found, maximizing torque at the beginning with a straighter body before bending to accelerate through the turn.

Our results differ from those of Howe & Astley (2020) in that we found discrete differences based on speed while they did not. We believe this was because all of the turns they studied would fall into our category of “fast turns”. They studied spontaneous turns, in which the fish turned through much smaller angles than those presented here. The largest in their data set was 120° (Howe & Astley, 2020), which is below the 180° turns we studied. However, even the turns that had a small angular change had high angular velocities, and would fall into our fast turn category. Their routine turns had maximum angular velocities of around 600°s^−1^, which is greater than the maximum angular velocities in our fast turns, which averaged around 430°s^−1^.

The slow turns we studied then may represent a qualitatively different behavior than fast turns in our data set, and than the turns studied by Howe & Astley (2020). We believe they represent the type of behavior that fish might use to maneuver in a controlled manner around an obstacle or while grazing. This type of behavior, though not widely studied in the lab, may be more representative of field behavior than much faster turns. For example, bluegill sunfish, studied in a pond in Massachusetts, almost never performed fast turns (Cathcart et al., 2017). Angular velocities were almost always below 40°s^−1^ (Cathcart et al., 2017), slower than many of our slow turns, though likely through a much smaller total angle. There are relatively few studies of the kind of slow controlled maneuver represented by slow turns here, but coelacanths make similar slow turns when maneuvering around an obstacle (Fricke & Hissmann, 1992). Fast turns are clearly important for avoiding predators or catching prey (Higham et al., 2025), but fish may not do them very often.

### Fast turns are not C-starts

Slow and fast turns show some key differences, but these differences are not because the fast turns are escape responses or C-starts. Escape responses are produced by a specialized neural circuit, which includes two giant neurons called Mauthner cells that activate red and white muscle all along one side of the body during the turn (Domenici & Hale, 2019). Even the fastest turns in our data set are slower than escape responses, and are likely produced by different neural circuits. The shortest turn in our study (0.6 s long) took much longer than the longest C-start (0.21 s) in Domenici & Blake’s (1997) review. The turns also had much lower maximum angular velocities, of around 600°s^−1^, compared to 2,000°s^−1^ or more in C-starts (also in bluegill sunfish; Tytell & Lauder (2008)). Moreover, in C-starts, fish tend to swing the head to one side (during the “C”) and back toward the other side as they kick out of the turn (Domenici & Blake, 1997), meaning that the head angular velocity has a positive and a negative peak. In the turns we measured, the fish only swung their heads to one side, meaning that angular velocity only had a positive peak (Fig. 3). During turning, bluegill in our study also reached a maximum snout linear velocity of 4.7 L/s, which is less than half of maximum that has been reported during bluegill C-starts (Webb, 1978).

### A diversity of turning patterns

We used the car to impose discrete differences in turning rates, which showed that some features of the turn vary continuously with speed, but that others, like the timing of torque and moment of inertia, seem to fall in separate categories. Therefore, more work could be done to determine exactly when a slow turn becomes a fast turn. Is the transition gradual or discrete? Future experimental work could address when this transition occurs for bluegill, and whether it differs for different species of fish.

Specifically, we studied a species thought to be specialized for maneuvering (Webb, 1984b), with a deep body and large pectoral fins. The tradeoff we highlighted between torque production and moment of inertia is likely to be different for different species, akin to how maneuverability and agility are different for different species. The tradeoff likely depends on both body shape and stiffness, but few studies have examined routine turning together with those parameters. Fish with relatively stiff bodies may find it challenging to bend to a high enough angle to reduce the moment of inertia for rapid turns. Indeed, a study of coral reef fishes found the lowest maneuvering performance in the deep-bodied foureye butterflyfish (*Chaetodon capistratus*) compared to the more elongate and likely more flexible bluehead wrasse (*Thalassoma bifasciatum*) (Gerstner, 2011), as long as they turned by bending their bodies. The butterflyfish were able to compensate and had very good maneuvering performance when they turned using their pectoral fins (Gerstner, 2011). Another study, however, found relatively little correlation between body shape and routine turning (Satterfield, Claverie & Wainwright, 2023). We suggest that this lack of correlation with body shape may be because the fish also vary in internal body mechanical properties such as stiffness, and stiffer-bodied fish do not curve their bodies as much to reduce moment of inertia as more flexible fishes (Jimenez et al., 2023).

In that case, it might be fish with stiffer bodies would not perform a kinematically distinct slow turn as we have defined it. The pattern of “planting” the tail as the snout begins the turn seems to be consistent across different fusiform species. Gray (1933) described a similar pattern in turning whiting *Gadus merlangus*. More recently, Thandiackal & Lauder (2020) found that the anterior and posterior body in zebrafish produce substantial positive work during turning, showing that they use the tail to drive the anterior body around through the turn.

More elongate and flexible fishes, such as eels and hagfishes, likely turn in a different way again. Our analysis was based on the idea that the body flexes in a single curve, and that the whole body rotates around a single center of rotation. For eels, their bodies are so flexible that they can pass a point or multiple points of flexion along the body so that different portions can rotate independently (Gray, 1933), which may circumvent the tradeoff that other less flexible fishes face. Many sharks also use very high body curvature during routine turns (Porter, Roque & Long Jr, 2009).

Finally, organisms like boxfish which are rigid (Walker, 2000) and rays which are laterally rigid (Parson, Fish & Nicastro, 2011) are thus less able to modulate their moments of inertia, potentially needing to find find alternate solutions to effectively turn around. However, despite their lateral rigidity, rays are still able to turn faster than other rigid bodied species of similar lengths (Fish et al., 2018). Furthermore, Dabiri et al. (2020) found similarities in the patterns even between zebrafish and jellyfish. Therefore, future studies could expand this speed dependent research to better understand and quantify how different morphologies allow fish or other aquatic organisms to resolve the tradeoff between moment of inertia and torque production.

## Conclusions

We used a novel device to elicit repeatable 180° turns in bluegill sunfish *Lepomis macrochirus* at two different turning rates. While many features of turns at different rates were similar, we found that bluegill seem to differ across turn speeds in how they prioritize producing torque and reducing angular moment of inertia (Fig. 11). At low speeds, they follow a similar pattern to turns in zebrafish (Dabiri et al., 2020) in which they maximized torque first and then minimized moment of inertia. At higher turning rates, they used the opposite order, minimizing moment of inertia before they maximized torque. Future work will be needed to identify whether these patterns hold for spontaneous turns over smaller angles, and how these patterns differ for fishes with different body shapes.

##  Supplemental Information

10.7717/peerj.21161/supp-1 Supplemental Information 1 Movie showing a slow turn from the below (top) and from the side (bottom)

10.7717/peerj.21161/supp-2 Supplemental Information 2 Movie showing a fast turn from the below (top) and from the side (bottom)

10.7717/peerj.21161/supp-3 Supplemental Information 3 ARRIVE checklist

## Additional Information and Declarations

### Field Study Permissions

The following information was supplied relating to field study approvals (*i.e.*, approving body and any reference numbers): We collected the fish under scientific collection permits approved by the Massachusetts Division of Fisheries and Wildlife (permit numbers 139.24SCF, 146.23SCF, 090.22SCF, and 174.20SCF).

### Data Availability

The following information was supplied regarding data availability: The code is available at Github and Zenodo: - https://github.com/andrewdclark/BluegillTurning
- Clark, A.D. & Tytell, E.D. (2026). andrewdclark/BluegillTurning: Data and Code for “Tradeoffs for bluegill sunfish turning at different rates” (v1.0). Zenodo. https://doi.org/10.5281/zenodo.19102452.

## References

[ref-1] Bandyopadhyay PR (2002). Maneuvering hydrodynamics of fish and small underwater vehicles. Integrative and Comparative Biology.

[ref-2] Bates D, Mächler M, Bolker B, Walker S (2015). Fitting linear mixed-effects models using Lme4. Journal of Statistical Software.

[ref-3] Cathcart K, Shin SY, Milton J, Ellerby D (2017). Field swimming performance of bluegill sunfish, *Lepomis macrochirus*: implications for field activity cost estimates and laboratory measures of swimming performance. Ecology and Evolution.

[ref-4] Clark A, Tytell E (2025). Fast turns are not sped up slow turns: how bluegill sunfish change their kinematics with turn speed. BioRxiv.

[ref-5] Dabiri JO, Colin SP, Gemmell BJ, Lucas KN, Leftwich MC, Costello JH (2020). Jellyfish and fish solve the challenges of turning dynamics similarly to achieve high maneuverability. Fluids.

[ref-6] Domenici P, Blake RW (1997). The kinematics and performance of fish fast-start swimming. Journal of Experimental Biology.

[ref-7] Domenici P, Hale ME (2019). Escape responses of fish: a review of the diversity in motor control, kinematics and behaviour. Journal of Experimental Biology.

[ref-8] Downs AM, Kolpas A, Block BA, Fish FE (2023). Multiple behaviors for turning performance of Pacific bluefin tuna (*Thunnus Orientalis*). Journal of Experimental Biology.

[ref-9] Drucker EG, Lauder GV (2000). A hydrodynamic analysis of fish swimming speed: wake structure and locomotor force in slow and fast labriform swimmers. Journal of Experimental Biology.

[ref-10] Drucker EG, Lauder GV (2001). Wake dynamics and fluid forces of turning maneuvers in sunfish. Journal of Experimental Biology.

[ref-11] Dunnington D, Thorne B, Hernangómez D (2023). https://cran.r-project.org/web/packages/ggspatial/index.html.

[ref-12] Eklund A, Trimble J (2021). https://cran.r-project.org/web/packages/beeswarm/index.html.

[ref-13] Ellerby DJ, Berlin CG, Cathcart KJ, Dornon MK, Feldman A, Gee JK, Moran CJ (2018). Assessing the ecological relevance of swimming performance traits: a case study of bluegill sunfish (*Lepomis macrochirus*). Aquatic Ecology.

[ref-14] Fath MA, Nguyen SV, Donahue J, McMenamin SK, Tytell ED (2023). Static stability and swim bladder volume in the bluegill sunfish (*Lepomis Macrochirus*). Integrative Organismal Biology.

[ref-15] Feldmeth CR (1983). Costs of aggression in trout and pupfish. Behavioral energetics.

[ref-16] Fish FE (2020). Advantages of aquatic animals as models for bio-inspired drones over present AUV technology. Bioinspiration & Biomimetics.

[ref-17] Fish FE, Kolpas A, Crossett A, Dudas MA, Moored KW, Bart-Smith H (2018). Kinematics of swimming of the manta ray: three-dimensional analysis of open-water maneuverability. Journal of Experimental Biology.

[ref-18] Fox J, Weisberg S (2019). An R companion to applied regression.

[ref-19] Fricke H, Hissmann K (1992). Locomotion, fin coordination and body form of the living coelacanth *Latimeria Chalumnae*. Environmental Biology of Fishes.

[ref-20] Fuiman LA, Webb PW (1988). Ontogeny of routine swimming activity and performance in zebra danios (*Teleostei: Cyprinidae*). Animal Behaviour.

[ref-21] Garrido-Jurado S, Muñoz Salinas R, Madrid-Cuevas FJ, Marıń Jiménez MJ (2014). Automatic generation and detection of highly reliable fiducial markers under occlusion. Pattern Recognition.

[ref-22] Gerstner CL (2011). Maneuverability of four species of coral-reef fish that differ in body and pectoral-fin morphology. Canadian Journal of Zoology.

[ref-23] Gray J (1933). Directional control of fish movement. Proceedings of the Royal Society B: Biological Sciences.

[ref-24] Hawkins OH, Di Santo V, Tytell ED, Higham TE, Lauder GV, Farrell AP, Brauner CJ, Eliason EJ (2025). Biomechanics and energetics of swimming. Integrative fish biomechanics, fish physiology.

[ref-25] Higham TE (2007). Feeding, fins and braking maneuvers: locomotion during prey capture in centrarchid fishes. Journal of Experimental Biology.

[ref-26] Higham TE, Gerringer ME, Axlid EG, Liao JC, Higham TE, Lauder GV (2025). Chapter 8—ecology and biomechanics of locomotion and feeding in fishes. Fish physiology.

[ref-27] Hitchcock AC, Chen T, Connolly E, Darakananda K, Jeong J, Quist A, Robbins A, Ellerby DJ (2015). Trade-offs between performance and variability in the escape responses of bluegill sunfish (*Lepomis Macrochirus*). Biology Open.

[ref-28] Howe SP, Astley HC (2020). The control of routine fish maneuvers: connecting midline kinematics to turn outcomes. Journal of Experimental Zoology Part A: Ecological and Integrative Physiology.

[ref-29] Jayne BC, Lauder GV (1993). Red and white muscle activity and kinematics of the escape response of the bluegill sunfish during swimming. Journal of Comparative Physiology. A, Sensory, Neural, and Behavioral Physiology.

[ref-30] Jimenez YE, Brainerd EL (2021). Motor control in the epaxial musculature of bluegill sunfish in feeding and locomotion. Journal of Experimental Biology.

[ref-31] Jimenez YE, Lucas KN, Long JH, Tytell ED (2023). Flexibility is a hidden axis of biomechanical diversity in fishes. Journal of Experimental Biology.

[ref-32] Karashchuk P, Rupp KL, Dickinson ES, Walling-Bell S, Sanders E, Azim E, Brunton BW, Tuthill JC (2021). Anipose: a toolkit for robust markerless 3D pose estimation. Cell Reports.

[ref-33] Kay M (2024). Ggdist: visualizations of distributions and uncertainty in the grammar of graphics. IEEE Transactions on Visualization and Computer Graphics.

[ref-34] Kumle L, Võ ML-H, Draschkow D (2021). Estimating power in (generalized) linear mixed models: An open introduction and tutorial in R. Behavior Research Methods.

[ref-35] Lecheval V, Jiang L, Tichit P, Sire C, Hemelrijk CK, Theraulaz G (2018). Social conformity and propagation of information in collective u-turns of fish schools. Proceedings of the Royal Society B: Biological Sciences.

[ref-36] Lenth RV, Banfai B, Bolker B, Buerkner P, Giné-Vázquez I, Herve M, Jung M, Love J, Miguez F, Piaskowski J (2025). https://cran.r-project.org/web/packages/emmeans/index.html.

[ref-37] Lucas KN, Lauder GV, Tytell ED (2020). Airfoil-like mechanics generate thrust on the anterior body of swimming fishes. Proceedings of the National Academy of Sciences, USA.

[ref-38] Lüdecke D, Ben-Shachar MS, Patil I, Waggoner P, Makowski D (2021). performance: an R package for assessment, comparison and testing of statistical models. Journal of Open Source Software.

[ref-39] Marcoux TM, Korsmeyer KE (2019). Energetics and behavior of coral reef fishes during oscillatory swimming in a simulated wave surge. Journal of Experimental Biology.

[ref-40] Parson JM, Fish FE, Nicastro AJ (2011). Turning performance of batoids: limitations of a rigid body. Journal of Experimental Marine Biology and Ecology.

[ref-41] Pereira TD, Tabris N, Matsliah A, Turner DM, Li J, Ravindranath S, Papadoyannis ES, Normand E, Deutsch DS, Wang ZY, McKenzie-Smith GC, Mitelut CC, Castro MD, D’Uva J, Kislin M, Sanes DH, Kocher SD, Wang SS-H, Falkner AL, Shaevitz JW, Murthy M (2022). SLEAP: a deep learning system for multi-animal pose tracking. Nature Methods.

[ref-42] Porter ME, Roque CM, Long JH (2011). Swimming fundamentals: turning performance of leopard sharks (*Triakis Semifasciata*) is predicted by body shape and postural reconfiguration. Zoology.

[ref-43] Porter ME, Roque CM, Long Jr JH (2009). Turning maneuvers in sharks: predicting body curvature from axial morphology. Journal of Morphology.

[ref-44] Rieucau G, Boswell KM, De Robertis A, Macaulay GJ, Handegard NO (2014). Experimental evidence of threat-sensitive collective avoidance responses in a large wild-caught herring school. PLOS ONE.

[ref-45] Roche DG, Tytell ED, Domenici P (2023). Kinematics and behaviour in fish escape responses: guidelines for conducting, analysing and reporting experiments. Journal of Experimental Biology.

[ref-46] Satterfield DR, Claverie T, Wainwright PC (2023). Body shape and mode of propulsion do not constrain routine swimming in coral reef fishes. Functional Ecology.

[ref-47] Schindelin J, Arganda-Carreras I, Frise E, Kaynig V, Longair M, Pietzsch T, Preibisch S, Rueden C, Saalfeld S, Schmid B, Tinevez J-Y, White DJ, Hartenstein V, Eliceiri K, Tomancak P, Cardona A (2012). Fiji: an open-source platform for biological-image analysis. Nature Methods.

[ref-48] Shadwick RE, Gemballa S, Shadwick RE, Lauder GV (2006). Structure, kinematics, and muscle dynamics in undulatory swimming.

[ref-49] Smith GD (1986). Numerical solution of partial differential equations: finite difference methods.

[ref-50] Thandiackal R, Lauder GV (2020). How zebrafish turn: analysis of pressure force dynamics and mechanical work. Journal of Experimental Biology.

[ref-51] Tiffan KF, Kock TJ, Haskell CA, Connor WP, Steinhorst RK (2009). Water velocity, turbulence, and migration rate of subyearling fall chinook salmon in the free-flowing and impounded snake river. Transactions of the American Fisheries Society.

[ref-52] Tytell ED, Farrell AP (2011). Experimental hydrodynamics. Encyclopedia of fish physiology: from genome to environment.

[ref-53] Tytell ED, Lauder GV (2008). Hydrodynamics of the escape response in the bluegill sunfish, *Lepomis macrochirus*. Journal of Experimental Biology.

[ref-54] Walker JA (2000). Does a rigid body limit maneuverability?. Journal of Experimental Biology.

[ref-55] Walker JA, Ghalambor C, Griset OL, McKenney D, Reznick D (2005). Do faster starts increase the probability of evading predators?. Functional Ecology.

[ref-56] Webb PW (1978). Fast-start performance and body form in seven species of teleost fish. Journal of Experimental Biology.

[ref-57] Webb PW (1983). Speed, acceleration and manoeuvrability of two teleost fishes. Journal of Experimental Biology.

[ref-58] Webb PW (1984a). Form and function in fish swimming. Scientific American.

[ref-59] Webb PW (1984b). Body and fin form and strike tactics of four teleost predators attacking fathead minnow (*Pimephales promelas*). Canadian Journal of Fisheries and Aquatic Sciences.

[ref-60] Webb PW (1986). Effect of body form and response threshold on the vulnerability of four species of teleost prey attacked by largemouth bass (*Micropterus salmoides*). Canadian Journal of Fisheries and Aquatic Sciences.

[ref-61] Webb PW (1991). Composition and mechanics of routine swimming of rainbow trout, *Oncorhynchus mykiss*. Canadian Journal of Fisheries and Aquatic Sciences.

[ref-62] Webb PW (2005). Stability and maneuverability.

[ref-63] Weihs D, Keyes RS, Stalls DM (1981). Voluntary swimming speeds of two species of large carcharhinid sharks. Copeia.

[ref-64] Wickham H, Averick M, Bryan J, Chang W, McGowan LD, François R, Grolemund G, Hayes A, Henry L, Hester J, Kuhn M, Pedersen TL, Miller E, Bache SM, Müller K, Ooms J, Robinson D, Seidel DP, Spinu V, Takahashi K, Vaughan D, Wilke C, Woo K, Yutani H (2019). Welcome to the Tidyverse. Journal of Open Source Software.

